# Etodolac Containing Topical Niosomal Gel: Formulation Development and Evaluation

**DOI:** 10.1155/2016/9324567

**Published:** 2016-07-11

**Authors:** Gyati Shilakari Asthana, Abhay Asthana, Davinder Singh, Parveen Kumar Sharma

**Affiliations:** Department of Pharmaceutics, M. M. College of Pharmacy, Maharishi Markandeshwar University, Mullana, Ambala, Haryana 133207, India

## Abstract

The present study aimed to investigate the delivery potential of Etodolac (ETD) containing topical niosomal gel. Niosomal formulations were prepared by thin film hydration method at various ratios of cholesterol and Span 60 and were evaluated with respect to particle size, shape, entrapment efficiency, and* in vitro* characteristics. Dicetyl phosphate (DCP) was also added in the niosomal formulation. Mean particle size of niosomal formulation was found to be in the range of 2 *μ*m to 4 *μ*m. Niosomal formulation N_2_ (1 : 1) ratio of cholesterol and surfactant displayed good entrapment efficiency (96.72%). TEM analyses showed that niosomal formulation was spherical in shape. Niosomal formulation (N_2_) displayed high percentage of drug release after 24 h (94.91) at (1 : 1) ratio of cholesterol : surfactant. Further selected niosomal formulation was used to formulate topical gel and was characterized with respect to its various parameters such as pH, viscosity, spreadability,* ex vivo* study, and* in vivo* potential permeation.* Ex vivo* study showed that niosomal gel possessed better skin permeation study than the plain topical gel. Further* in vivo* study revealed good inhibition of inflammation in case of topical niosomal gel than plain gel and niosomal formulation. The present study suggested that topical niosomal gel formulations provide sustained and prolonged delivery of drug.

## 1. Introduction

Etodolac (ETD) is a nonsteroidal anti-inflammatory drug (NSAID) of selective COX-2 inhibitor class used for osteoarthritis and rheumatoid arthritis. Its therapeutic effects are due to its ability to inhibit prostaglandin synthesis (“as discussed by Inoue et al. [1]”). Prostaglandins are the chemicals which are responsible for pain and the fever and tenderness that occur with inflammation. It possesses poor solubility and also undergoes extensive first pass metabolism that could be the reason of its low bioavailability. Its terminal half life is 7 hrs and hence frequent dosing is required. Thus, to avoid aforementioned issues of ETD, it is desirable to encapsulate the drug in the vesicular system to prolong the existence of the drug in systemic circulation and perhaps increase the bioavailability. Continuous oral use of ETD causes serious gastrointestinal disturbance such as ulcer, stomach, or intestinal bleeding and chest pain which can be fatal. To avoid such problem there are other alternatives like coating of polymer that avoid release of drug molecule in gastrointestinal tract (GIT). Another approach to overcome such problem is changing the route of drug administration, namely, buccal route, topical route, nasal route, transdermal route, and so forth. Topical route is most preferable as easy to apply and provide high level of patient satisfaction. Transdermal route also avoid first pass metabolism associated with conventional route of drug administration. It also provides easy elimination of drug in case of toxicity and reduced fluctuation in plasma drug concentration level and thus decreased side effect. Various novel drug delivery technologies have been applied. Various novel delivery technologies including ethosomes (“as discussed by Chintala and Padmapreetha [2]”), microsphere (“as discussed by Kumar et al. [3]”), thermogels for rectal delivery (“as discussed by Barakat [4]”), and hydrophilic gel (“as discussed by Tas et al. [5]”) have been reported for delivery of Etodolac.

Topical drug delivery means the application of drug to skin for localized effect. The skin is one of the most widespread and freely available structures of the human body. Skin of an average adult body covers a surface approximately 2 m^2^ and receives about one-third of the blood circulating through the body (“as discussed by Sharma et al. [6]”). It acts as a regulator in retaining the body heat, plays a part in regulation of blood pressure, and protects against the penetration of ultraviolet rays. Dermal drug delivery has number of advantages like longer duration of action, dosing flexibility, reduced side effects, uniform plasma levels, high patient compliance, and so forth but it has some disadvantages like possibility of local irritation effect, erythema, itching, and low permeability of drugs in the stratum corneum (“as discussed by Bhowmik et al. [7]”). Stratum corneum is outermost and top layer of epidermis which is impermeable to water and behaves like tough flexible membrane. It contains dead keratinized cells called corneocytes. For the permeation of drug to this barrier many technologies and systems have been investigated and one of the most promising techniques is the vesicular carrier for drug delivery through the skin.

Novel drug delivery carriers have great potential for dermal delivery. The lipidic and nonlipidic vesicular systems like liposome, transfersome, ethosome, and niosome are used to overcome the problem associated with topical conventional formulation. Drug delivery system using novel vesicular carrier, such as liposome or niosome, has distinct advantages over microspheres, nanoparticles, and other carriers in terms of better entrapment of drugs (payload characteristics), target site specificity, and handling premature drug release (burst effect). In 1985, niosomes were studied as an alternative to liposome because they offer some benefits over liposome such as being more stable, nontoxic, and economic due to low cost of nonionic surfactant as compared to phospholipids which are prone to oxidation. Incorporation of surfactants within niosomes may also enhance the efficacy of the drug, possibly by facilitating its uptake by the target cells. Niosomes are biodegradable, biocompatible, relatively nontoxic, and an alternative of liposome. They can be utilized in the delivery of wide variety of drugs as it has capability to entrap hydrophilic, lipophilic, and amphiphilic drugs. For transdermal route of administration NSAIDs, hormone, antibacterial, and antifungal drugs are most preferably used.

The objectives of the present study were to develop ETD loaded niosomal gel for transdermal delivery and to provide prolonged and sustained release of drug, so that required dose is decreased, and to avoid GI side effect and first pass hepatic metabolism.

## 2. Materials and Methods

### 2.1. Materials

Etodolac (ETD) was received as gift sample from IPCA Laboratories Ltd., Mumbai. Chloroform, acetone, potassium dihydrogen orthophosphate, sodium chloride, sodium hydroxide, methanol, Disodium hydrogen phosphate, and dialysis membrane were obtained from Himedia. Cholesterol, dicetyl phosphate (DCP), and Span 60 were taken from Sigma-Aldrich.

### 2.2. Preparation of Niosomal Formulation

In the present study, niosomal formulations were prepared by thin film hydration technique as reported earlier with slight modifications (“as discussed by Balakrishnan et al. [8]”) by using various ratios of Span 60 and cholesterol as shown in Table 1. DCP (5 mg) was added in the formulation to keep the niosomal formulation stable for long period of time. Accurately weighted quantities of surfactants and cholesterol were taken to give the desired ratio and were dissolved in 10 mL chloroform in a round bottom flask and DCP was added to the above mixture. Then accurately weighed amount of drug was added into the solvent. The solvent was evaporated in a rotary flash evaporator under a vacuum of 20 inches of Hg at a temperature of 60°C at 120 rpm until a smooth, dry lipid film was obtained. Then film was hydrated with 10 mL of phosphate buffer saline (PBS) pH 7.4 for 3 hr at 60 ± 2°C with shaking on a water bath. The niosomal suspension was kept at 2–8°C for 24 hr. Developed niosomal formulation was evaluated with respect to particle size, shape, entrapment efficiency, and* in vitro* drug release profile.

## 3. Evaluation of Niosomal Formulation

Niosomes formulations were characterized with respect to shape, particle size distribution, entrapment efficiency, and* in vitro* release studies.

### 3.1. Particle Shape and Morphology

**Figure 1:**
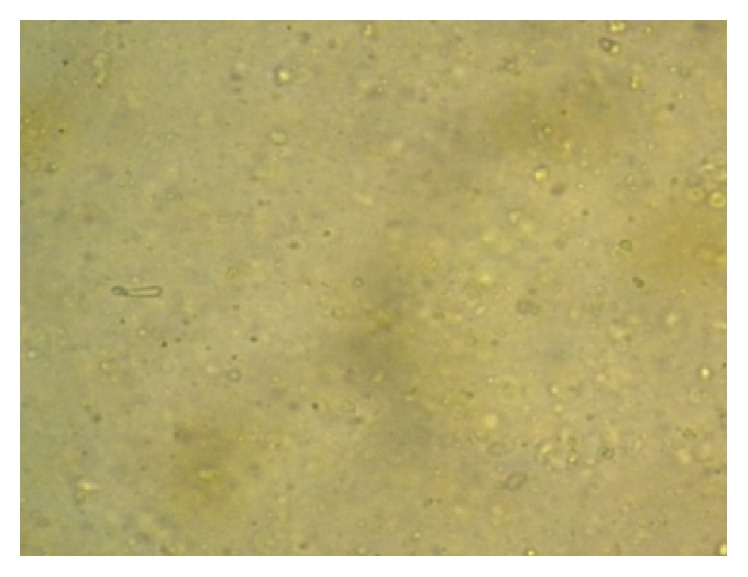
Optical microscopy of niosomal formulation at 45x.

**Figure 2:**
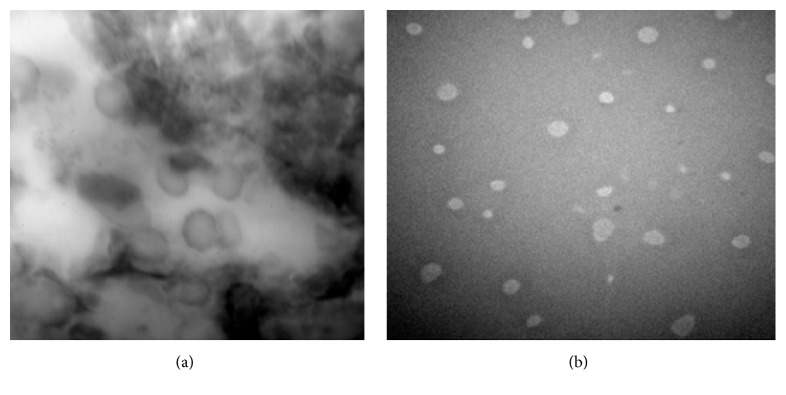
TEM image of N_2_ formulation at (a) 60,000x and (b) 18,000x.

Shape and morphology of niosomal formulations were determined by optical microscopy and Transmission Electron Microscopy (TEM) and result were shown in Figures 1 and 2.

### 3.2. Particle Size

The particle size of the niosomal suspension was determined by optical microscopy. A drop of niosomal suspension was placed on a glass slide. A cover slip was placed over the niosomes suspension and evaluated the average vesicle size by an ordinary optical microscope using a precalibrated ocular eye piece micrometer (“as discussed by Firthouse et al. [9]”).

### 3.3. Entrapment Efficiency

Entrapment efficiency of niosomal formulations was determined by centrifugation method. 10 mL niosomal suspension was poured into a stopper test tube and centrifuged by using cooling centrifuge at 10,000 rpm maintained at 4°C for 90 minutes and then filtered by using Whatman filter paper to obtain clear fraction. The clear fraction was used for the determination of free drug by using UV spectrophotometer at 278 nm (“as discussed by Sathali and Sangeetha [10]”). The encapsulation efficiency was calculated using the formula

(1) $$\begin{array}{c} \text{Entrapment Efficiency}\left(\;\%\;\right)=\left[\;\frac{\left(C_{t}-C_{f}\right)}{C_{t}}\times 100\;\right], \end{array}$$

where *C*_*t*_ is concentration of total drug and *C*_*f*_ is concentration of unentrapped drug.

### 3.4. *In Vitro* Release Studies

*In vitro* release studies of niosomes suspension were carried out by dialysis bag method (“as discussed by Sathali and Rajalakshmi [11]”). A dialysis sac was washed and kept in distilled water for soaking. The vesicle suspension was pipetted into a bag made up of tubing and sealed followed by placing the dialysis bag into a beaker containing 500 mL of PBS pH 7.4. The beaker was placed over magnetic stirrer (50 rpm) and the temperature was maintained at 37°C ± 0.5°C. Samples were withdrawn at predetermined time intervals. Sink condition was maintained throughout the experiment by replacing the withdrawing sample with fresh medium. Samples were diluted and analysed for drug content by using UV/visible spectrophotometer at 278 nm (“as discussed by Sathali and Rajalakshmi [11]”).

### 3.5. Formulation and Evaluation of Gels

Gel base was prepared by dispersing 1% w/w Carbopol 940 in distilled water and then was allowed to swell for 1 hour. After that glycerin was added to the dispersion with continuous homogenization. The pH was adjusted by triethanolamine (“as discussed by Solanki et al. [12]”).

### 3.6. Preparation of Plain Topical Gel

Plain gel was prepared by adding drug (2% w/w) in 1% w/w Carbopol 940 base for the comparison with niosomal gel (N_2_G).

### 3.7. Preparation of Niosomal Gel

The measured volume of N_2_ niosomal formulation was centrifuged by using cooling centrifuge for 90 mins at 4°C and 12,000 rpm. The semisolid mass of niosomes was separated from supernatant and mixed in the 1% Carbopol gel base by using electric homogenizer (“as discussed by Solanki et al. [12]”).

### 3.8. Evaluation of Topical Gel

The niosomal gel and plain topical ETD gel were characterized with respect to pH, viscosity, spreadability, and* ex vivo* permeation studies.

### 3.9. pH Measurements

The pH of the gel formulations was delivered by using digital pH meter. Before measurement pH meter was calibrated and readings were taken by dipping the glass rod into the gel formulations (“as discussed by Jain et al. [13]”).

### 3.10. Viscosity Measurement

The viscosity of gel formulations was determined by Brookfield viscometer. 25.0 g gel was taken in beaker and spindle number 4 was rotated at 50 rpm and viscosity of the sample was determined (“as discussed by Jain et al. [13]”).

### 3.11. Spreadability

The spreadability of gel formulations was determined by using spreadability apparatus. 1.0 g of gel sample was placed on the lower slide and upper slide was placed on the top of the sample (“as discussed by Jain et al. [13]”). The spreadability was determined by the formula

(2) $$\begin{array}{c} S=\frac{m\times l}{t}, \end{array}$$

where *S* is spreadability, *m* is weight tied to upper slide, *l* is length travel by upper slide, and *t* is time.

### 3.12. *Ex Vivo* Permeation Studies

The* ex vivo* permeation studies of plain topical gel and niosomal gel were carried out with Franz diffusion (FD) cell using dehaired rat skin followed by hydration for 30 minutes in PBS pH 7.4 at room temperature to remove the extraneous debris and leachable enzymes. Then excised skin was placed between donor and receptor compartments of the FD cell. Gel was placed in donor compartment and PBS pH 7.4 was taken in receptor compartments as media. Temperature of cell was maintained at 37°C ± 0.5°C. The assembly was kept on magnetic stirrer and samples were withdrawn at time intervals of 1, 2, 3, 4, 6, 8, 12, and 24 h and replaced with equal volume of fresh media. Samples were analyzed by UV spectrophotometer at 278 nm and cumulative % of drug release was calculated (“as discussed by Srikanth et al. [14]”).

### 3.13. Stability Studies

Niosomal formulation was selected on the basis of entrapment efficiency and* in vitro* release studies. Stability studies were assessed by keeping N_2_ niosomal suspension and niosomal gel in sealed glass vials and storing them in two different storage conditions, that is, refrigeration temperature and room temperature for a period of 30 days. The samples were withdrawn at different time intervals over a period of one month and the residual content was determined spectrophotometrically (“as discussed by Arora et al. [15]”).

### 3.14. *In Vivo* Studies

White Albino rats of either sex weighing between 180 and 200 g were selected for study. The approval of the Institutional Animal Ethical Committee CPCEA No. MMCP/IAEC/12/8 was obtained before starting the study. The animals were divided into four groups, each consisting of six rats. Groups II, III, and IV were subjected to topical gel of ETD drug, niosomal formulation, and topical niosomal gel formulation, respectively, whereas Group I was treated as control. Anti-inflammatory activities of the drug by developed formulations were determined by using paw edema method on the basis of the inhibition of the volume of the hind paw edema induced by phlogistic agent. For present study 1% w/v carrageenan solution in 0.9% w/v was used as phlogistic agent.

### 3.15. Anti-Inflammatory Activity

The animals were fasted overnight and all groups were treated by applying the formulations containing drug equivalent to 20 mg/kg body weight on the left paw of the rats, with respective gels. The area of application was covered with bandages and it was left in place for 3 hrs. The dressing was then removed and the gel remaining on the surface was wiped off with cotton. The animals were then injected with 0.1 mL of 1% w/v of carrageenan solution in plantar region of left hind paw and the paw volume was measured after 1 hr, 2 hr, 3 hr, 4 hr, 5 hr, and 6 hr and 8 hr, using modified or specially designed assembly which works on the Archimedes principle. The right paw served as a reference of noninflamed paw for comparison. The percentage difference between right and left paw volumes was taken as percent edema produced (“as discussed by Shinde and Kanojiya [16]”). The percent edema produced with test samples was subtracted from percent edema produced in control group to obtain percent edema inhibition by respective groups. Percent inhibition of edema is directly proportional to the anti-inflammatory activity.

## 4. Result and Discussion

### 4.1. Evaluation of Niosomal Formulation

Developed niosomal formulations were characterized with respect to particle size, shape, entrapment efficiency, and* in vitro* drug release profile.

#### 4.1.1. Shape and Morphology

Shape and morphology of niosomal formulations were determined by optical microscopy. It was clearly observed from Figure 1 that niosomes are spherical in shape.

#### 4.1.2. Transmission Electron Microscopy (TEM)

Morphological characteristics of niosomal formulations were further confirmed by TEM analysis. TEM photomicrograph of (N_2_) niosomal formulation at 60,000x and 18,000x magnification revealed the spherical shape of niosomes. Further it was observed from the TEM images that niosomes are hollow vesicular structure.

#### 4.1.3. Particle Size

**Figure 3:**
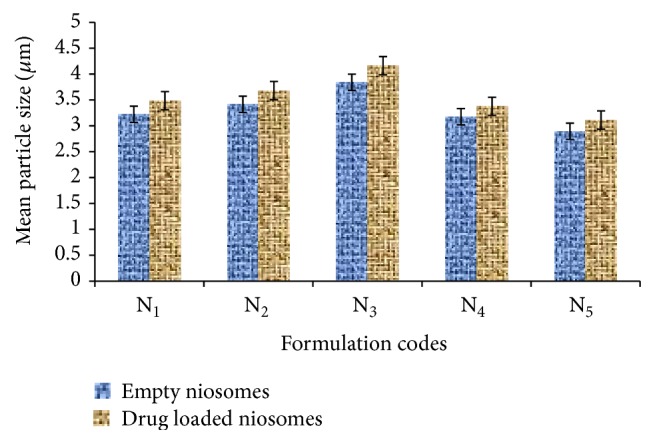
Particle size of niosomes containing Span 60.

Particle size of niosomal formulation at different ratio of cholesterol and surfactant was shown in Figure 3. Mean particle size of the niosomal formulation was found to be in the range of 2 *μ*m to 4 *μ*m. It was clearly depicted from Figure 3 that particle size of niosomal formulations was increased on increasing the cholesterol content. Cholesterol content provides strength to the nonpolar tail of nonionic surfactant. At low cholesterol content, it is to be expected that the cholesterol and nonionic surfactant are in close packing with increasing curvature and reducing size. As the cholesterol content increases, it would reduce the content of surfactants and also increased the hydrophobicity of bilayer membrane thus increasing vesicles radius in a way to establish more thermodynamic stable form. Rigid structure of bilayer membrane due to cholesterol content also provides resistance to reduce size due to sonication and results in vesicles with bigger size.

#### 4.1.4. Entrapment Efficiency

**Figure 4:**
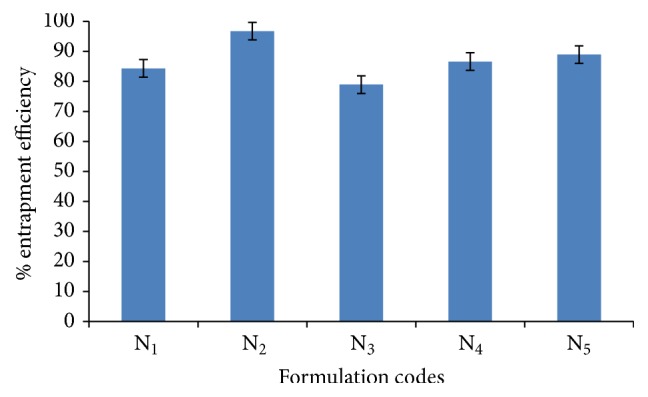
Entrapment efficiencies of various niosomal formulations containing Span 60.

Entrapment efficiency of various niosomal formulations was determined at different ratio of cholesterol and surfactant by centrifugation method and result was shown in Figure 4. It was clearly depicted from the data shown in Figure 4 that entrapment efficiency of the niosomal formulation was increased on increasing the cholesterol ratio from 0.5 to 1 whereas on further increase in cholesterol ratio from 1 to 1.5 the entrapment efficiency was decreased. There could be two factors for such result: first, with increase of cholesterol ratio, hydrophobicity and stability of bilayers vesicles increase and permeability decreases which may lead to efficiently trapping the hydrophobic drug into bilayers as the vesicles formed. Secondly higher amount of cholesterol may compete with the drug for packing space within the bilayer, hence excluding the drug as the amphiphiles assembled into drugs (“as discussed by Balakrishnan et al. [8]”).

Entrapment efficiency of niosomes was also influenced by varying the amount of surfactant. As the amount of surfactant in niosomal formulation increased, the decrease in leakage of drug results in enhancement of entrapment efficiency (“as discussed by Salih et al. [17]”).

#### 4.1.5. *In Vitro* Studies

**Figure 5:**
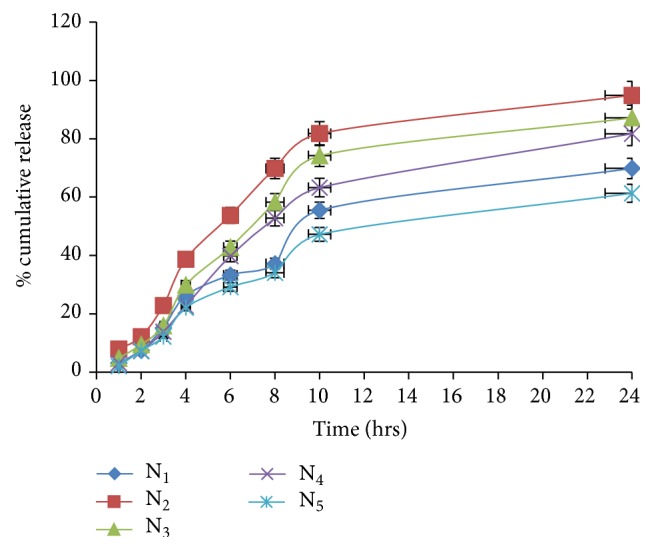
*In vitro* release profile of niosomal formulations.

*In vitro* studies of niosomal formulations were carried out in PBS pH 7.4 by dialysis method on magnetic stirrer and results were shown in Figure 5 (see Table 1 of Supplementary Material available online at http://dx.doi.org/10.1155/2016/9324567). It was observed from the data that* in vitro* drug release of niosomal formulation was sharply increased up to 24 hr. It was found that N_2_ formulation displayed maximum drug release that is 94.91% after 24 hr at 1 : 1 ratio of cholesterol to surfactant. Amount of surfactant varied in niosomal formulation affects the release profile of drug. As the ratio of surfactant increases from 1 : 1 to 1 : 1.5 (cholesterol : surfactant), the drug release from niosomal vesicles was decreased. This could be due to the fact that the increase in surfactant ratio acts as depot and reduces leakage of drug from niosomes to dissolution media (“as discussed by Salih et al. [17]”).

On the basis of the above result it was found that N_2_ possesses highest entrapment efficiency and* in vitro* release profile; thus it was selected for further study.

#### 4.1.6. Characterization of Niosomal Gel Formulations

*(1) Physical Examination.* Gels were examined for physical appearance and results of the physical examination of the gel were shown in Table 2.

Plain gel and niosomal gel formulations were white in color, were homogenous, and had smooth texture.

*(2) pH Measurement.* pH of the niosomal gel and plain gel as shown in Table 2 lied in the normal pH range of skin to avoid any risk of irritation upon the application to the skin.

*(3) Viscosity Measurements.* The viscosities of the gel formulations are optimum as they provide good* in vitro* release of drug through the gel and results were shown in Table 2. 

**Figure 6:**
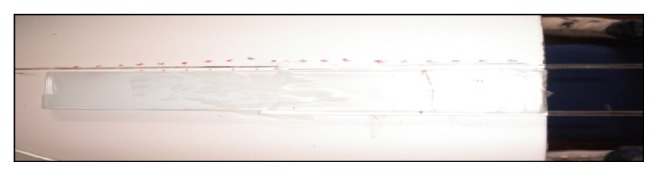
Spreadability studies.

*(4) Spreadability.* Spreadability of the plain gel and niosomal gel formulation were found to be better as compared to plain gel as shown in Table 2 and Figure 6. This could be because of loose gel matrix of gel due to presence of vesicles.

*(5) Ex Vivo Studies.* The* ex vivo* skin permeation study of niosomal gel and plain gel was carried out with Franz diffusion cell using abdominal skin of male Albino rat by using PBS (pH 7.4) as quality media.

**Figure 7:**
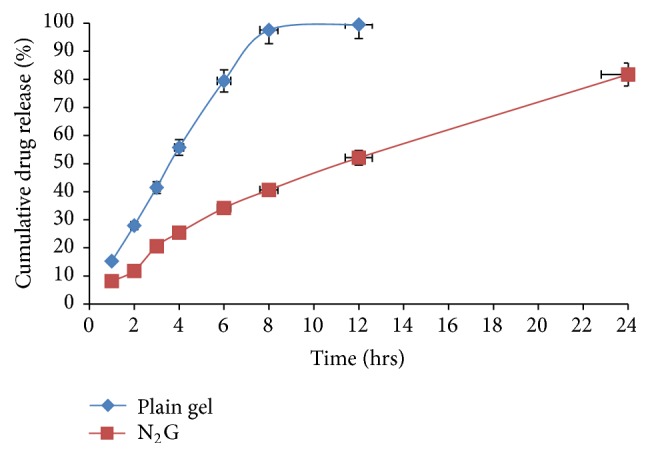
Comparative* ex vivo* permeation study of gel formulations.

It was displayed from the data as shown in Figure 7 that from drug release 15.28% of drug release was reported from ETD topical gel after 1 hr and drug release was significantly increased from 15.28% to 97.57% up to 8 hrs; thereafter only 1.91% of drug was released in next 4 hrs, whereas in case of topical niosomal gel only 8.13% of drug release was observed after 1 hr and continuous and appreciable increase in cumulative drug releases was reported up to 24 hrs. After 24 hrs only 81.73% of drug release was recorded. These results show that the slow and sustained release of drug through the rat skin for prolonged period of time was obtained in case of niosomal gel formulation as compared to plain gel to ETD. Faster release of the ETD from the plain gel may be due to free drug being present in gel structure as compared to niosomal gel in which drug was entrapped into niosomal vesicular structure. In case of niosomal gel, drug permeated due to either the fusion of the niosomes with the intercellular lipid of the stratum corneum, direct transfer of drug from niosomes to the skin, or the penetration enhancement effect of the nonionic surfactant of niosomes (“as discussed by Shilpa and Vijay [18]”).

**Figure 8:**
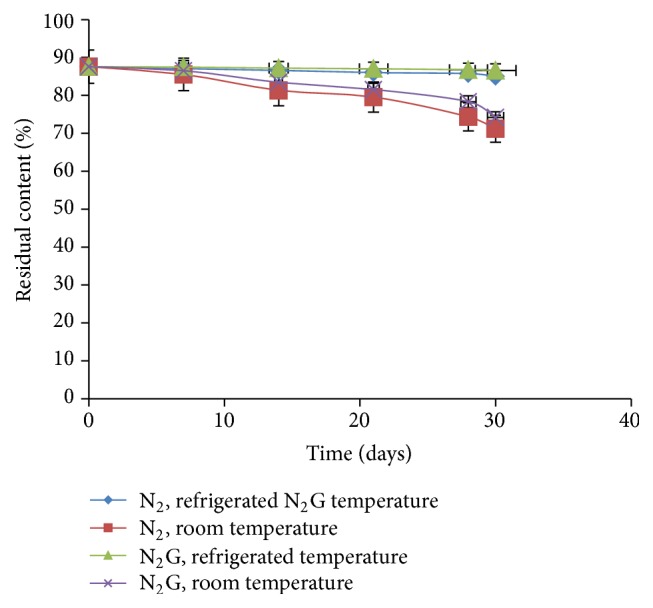
Stability studies of N_2_ and N_2_G formulation at different storage conditions.

*(6) Stability Studies.* Stability studies were carried out by keeping N_2_ formulation and niosomal gel formulation (N_2_G) for one month at different storage conditions. At different time points, sample was withdrawn and percent residual content was determined by UV spectrophotometer and results were shown in Figure 8.

It was observed from the results slight reduction in residual drug content at room temperature, whereas, in case of refrigerated temperature, no significant change was observed in residual content. Thus developed niosomal formulation was found to be stable at the end of the study on storage condition.

**Figure 9:**
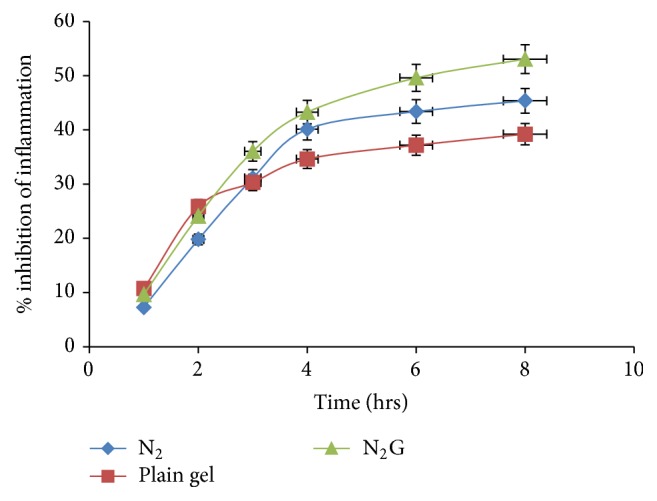
Comparative* in vivo* percentage edema inhibition.

*(7) In Vivo Studies. In vivo* anti-inflammatory activity was determined by using carrageenan induced rat paw edema method. The percentage inhibition of edema was calculated from different formulations and results were shown in Figure 9 and Table 2 of Supplementary Material.

Maximum% edema inhibition was observed with niosomal gel formulation as compared to the niosome suspension and plain gel after 8 hrs. Although the difference observed is narrow at end point, however, improvement is consistent at all time points, which presents the significance of outcome. According to both* ex vivo* and* in vivo* studies N_2_G gel showed the better permeation and effectiveness as compared to other formulations. This may be due to efficient hydration of thin film and more total amount of drug entrapped.

## 5. Conclusion

Niosomal formulation was successfully prepared by thin film hydration technique using different ratios of cholesterol and surfactant (Span 60) and dicetyl phosphate (DCP). It was found that niosomal formulation N_2_ having cholesterol : surfactant ratio (1 : 1) showed better entrapment efficiency and* in vitro* release profile. Selected niosomal formulation was further incorporated in Carbopol gel base to prepare niosomal gel. Both niosomal formulation and niosomal gel were found to be stable at different storage conditions. Niosomal gel was prepared, exhibiting better skin permeation study when compared with plain gel. Further inhibitions in inflammation by developed niosomal gel were also found maximum when compared to plain gel and niosomal formulation. The result of present research work reveals that niosomal gel formulation could be the better choice for delivery of wide verities of drug for transdermal administration.

## Supplementary Material

Table 1 displayed the *in vitro *drug release profile of the various niosomal formulations containing Etodolac. *In vitro *drug release from niosomal formulation was reported within the range of 61.31% to 94.91%. The highest drug release was found with Niosomal formulation (N_2_) at cholesterol: surfactant (1:1) ratio.
Table 2 shows the percent edema inhibition of various formulations including Niosomes, plain topical gel and topical niosomal gel containing Etodolac. The results revealed that the topical niosomal gel of Etodolac displayed maximum (53.07%) percent of edema inhibition compared to other formulations.

## Figures and Tables

**Table 1:** Composition of niosomal formulations at various ratios of cholesterol : Span 60.

Sr. number	Formulation codes	Drug : cholesterol : Span 60 (molar ratio)	Dicetyl phosphate (DCP) (mg)
1	N_1_	1 : 0.5 : 1	5.0
2	N_2_	1 : 1 : 1	5.0
3	N_3_	1 : 1.5 : 1	5.0
4	N_4_	1 : 1 : 0.5	5.0
5	N_5_	1 : 1 : 1.5	5.0

**Table 2:** Physical examination and other characteristics of plain and niosomal gel formulation.

Sr. number	Formulation codes	Color	Homogeneity	Texture	pH	Viscosity (cps)	Spreadability (gm·cm/sec)
1	N_2_G	White	Homogenous	Smooth	6.70 ± 0.05	6831 ± 9.47	18.25 ± 1.24
2	Plain gel	White	Homogenous	Smooth	6.60 ± 0.08	6502 ± 8.51	13.71 ± 0.83

## Abbreviations

ETD: Etodolac
DCP: Dicetyl phosphate
NSAID: Nonsteroidal anti-inflammatory drug
GIT: Gastrointestinal tract
TEM: Transmission Electron Microscopy
UV: Ultraviolet
RPM: Revolution per minute
FD: Franz diffusion.
