# Supplementary material for: Etodolac Containing Topical Niosomal Gel: Formulation Development and Evaluation
Source: J Drug Deliv. 2016 Jul 11;2016:9324567. doi: 10.1155/2016/9324567 (PMC4958486; doi:10.1155/2016/9324567)
Supplement: Supplementary file 1 — Table 1 displayed the in vitro drug release profile of the various niosomal formulations containing Etodolac. In vitro drug release from niosomal formulation was reported within the range of 61.31% to 94.91%. The highest drug release was found with Niosomal formulation (N2) at cholesterol: surfactant (1:1) ratio. Table 2 shows the percent edema inhibition of various formulations including Niosomes, plain topical gel and topical niosomal gel containing Etodolac. The results revealed that the topical niosomal gel of Etodolac displayed maximum (53.07%) percent of edema inhibition compared to other formulations. [file 9324567.f1.pdf]

***Table 1: In vitro* release data (Figure 5)**

| Time (Hrs) | $N_1$ | $N_2$ | $N_3$ | $N_4$ | $N_5$ |
|---|---|---|---|---|---|
| 1 | 3.16±0.21 | 7.95±0.41 | 4.83±0.23 | 3.11±0.24 | 2.14±0.17 |
| 2 | 7.53±0.27 | 12.18±0.38 | 9.42±0.36 | 7.22±0.22 | 7.37±0.34 |
| 3 | 15.17±0.39 | 22.87±0.11 | 15.89±0.44 | 13.92±0.19 | 12.19±0.22 |
| 4 | 26.21±0.25 | 38.73±0.31 | 29.93±0.12 | 22.67±0.31 | 22.17±0.18 |
| 6 | 33.29±0.12 | 53.75±0.26 | 42.78±0.27 | 39.88±0.16 | 29.19±0.24 |
| 7 | 37.19±0.17 | 69.82±0.13 | 58.29±0.11 | 52.77±0.23 | 34.09±0.39 |
| 8 | 55.49±0.11 | 81.79±0.19 | 74.19±0.17 | 63.28±0.14 | 47.23±0.41 |
| 9 | 69.83±0.24 | 94.91±0.21 | 87.15±0.14 | 81.73±0.33 | 61.31±0.26 |

**n=3**

Table 1 displayed the *in vitro* drug release profile of the various niosomal formulations containing Etodolac. *In vitro* drug release from niosomal formulation was reported within the range of 61.31% to 94.91%. The highest drug release was found with Niosomal formulation ($N_2$) at cholesterol: surfactant (1:1) ratio.

**Table2 : Percent edema inhibition (Figure 9)**

| Time (hrs) | N2 | Plain gel | N2G |
|---|---|---|---|
| 1 | 7.3±0.34 | 10.75±0.17 | 9.67±0.15 |
| 2 | 19.82±0.14 | 25.86±0.21 | 24.13±0.24 |
| 3 | 31.14±0.29 | 30.32±0.23 | 36.06±0.39 |
| 4 | 40.15±0.32 | 34.64±0.32 | 43.30±0.17 |
| 6 | 43.41±0.19 | 37.20±0.11 | 49.61±0.22 |
| 8 | 45.38±0.26 | 39.23±0.19 | 53.07±0.19 |

**n=6**

Table 2 shows the percent edema inhibition of various formulations including Niosomes, plain topical gel and topical niosomal gel containing Etodolac. The results revealed that the topical niosomal gel of Etodolac displayed maximum (53.07%) percent of edema inhibition compared to other formulations.
